# Nitric Oxide and Related Aspects Underlying Angina

**DOI:** 10.2174/1874192401711010033

**Published:** 2017-04-17

**Authors:** Carolina Baraldi Araujo Restini, Leticia Gonçalves

**Affiliations:** Biotechnology Dept. (Lab: Cardiorenal Pharmacology)/Medical School, University of Ribeirao Preto (UNAERP), Ribeirão Preto-SP, Brazil

**Keywords:** Angina, Cardiovascular symptoms, Cell signaling, Nitrergic nerves, Nitric Oxide, Nitric Oxide Synthase, Pain

## Abstract

Increased number of patients affected by metabolic syndrome (MS) has prompted the necessity of better understanding what is involved in such syndrome. Nevertheless, the establishment of promising therapies depends on the knowledge about the interaction of molecules within MS. In such context, Nitric Oxide (NO) emerges from a bulk of works relating its roles on aspects of MS, including cardiovascular diseases, their symptoms and comorbidities, which are thought to be triggered by similar sources. NO, nitric oxide synthase and enzymatic chains are keys for those disease and symptoms processes. NO has been separately described as part of hypertensive, ischemic and pain signaling. Although there are similar pathways likely shared for generating cardiovascular symptoms such angina, they are barely associated to NO in literature. The present review aims to clarify the patterns of NO alteration in metabolic syndrome directly concerned to cardiovascular symptoms, especially angina.

## 1 INTRODUCTION

Angina is the prominent symptom of coronary heart disease (CHD), a condition, among others that describes one of the main disorders related to metabolic syndrome (MS). This syndrome is accepted to be an association among obesity, hypertension, dyslipidemia, glucose metabolism alteration (glucose intolerance, insulin resistance or diabetes type II) and responsible for the higher risk of cardiovascular diseases [1]. Studies around the world have been observing individuals with MS presenting inferior prognosis and sharp mortality when comparing with non-MS patients [2-6].

Multicenter studies have demonstrated the Nitric Oxide (NO) influences in cardiovascular system. Even though hypertension, vascular diseases and metabolic syndrome have been related to important symptom such as angina, the absence or deviations in NO signaling are scarcely related to cardiovascular disease and/or their symptoms. The importance of this association consists of NO controlling relevant functions such as neurotransmission [7, 8], vascular tone [9, 10], gene transcription [9, 10], mRNA translation [11, 12] and post-translational modifications of proteins [13, 14]. Overall, despite the lacking of association in literature, NO plays a trivial role in angina.

The main damages involved on the NO signaling are related to oxidative stress and the development of the components predisposing MS and its symptoms. In the present review, we sought to clarify the patterns of NO alteration in metabolic syndrome directly concerned to cardiovascular symptoms, especially angina.

## 2 THE GENERATION OF NITRIC OXIDE

NO is produced from dietary sources *via* Nitrate-Nitrite-NO pathway or from endogenous turnover. The main differences between these pathways are basically the enzymes, the substrates, respectively nitrate and L-arginine, and the requirement of molecular oxygen (O_2_) for NO turnover pathway. Even though there are two processes, they are linked by the reduction of endogenously produced NO, which provides the largest endocrine source of directly bioavailable NO to inorganic nitrite (NO_2_^−^) [15].

The NO synthesis from dietary intake is dependent on xanthine oxidoreductase (XOR). Among other functions, XOR is a major NO_2_^−^ reductase enzyme linked to cellular NO signaling events [16-33] Fig. (**1**). This enzyme is essential for nitrate (NO_3_^−^) use from diet.

In other way, NO produced from L-arginine requires an enzyme called nitric oxide synthase (NOS). There are three classical isoforms: endothelial (eNOS), neuronal (nNOS) and inducible (iNOS). These isoenzymes are derived from different genes and trigger diverse organic processes [34]. eNOS and nNOS are constitutively expressed and are dependent on Ca^2+^ for activation. In contrast, iNOS is usually expressed in proinflammatory processes and Ca^2+^ independent [35-43]. Besides the classical, there is a novel NOS isoform, mitochondrial NOS (mtNOS), which is present in mitochondria [44-46] and appears to regulate cellular oxygen consumption/energy metabolism without engendering oxidative stress [47, 48]. Positive vascular effects are well established as mediated by cellular pathways of NOS/L-arginine NO signaling [35, 49].

**Fig. (2):**
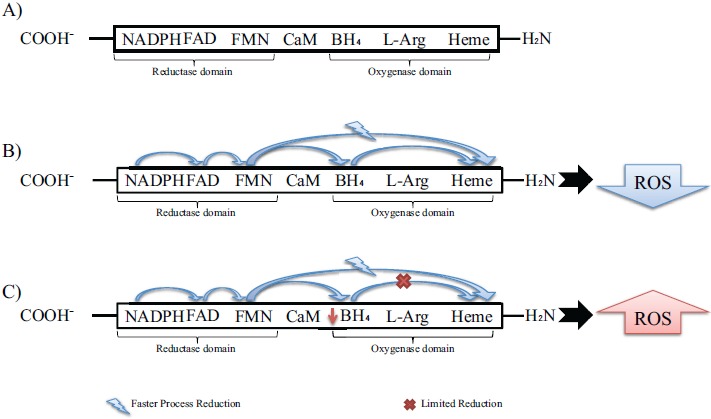
NOS isoforms and reactive oxygen species (ROS) production through electron flowing. A) NOS enzyme schematic structure with main cofactors. B) Normal electron flowing in NOS enzyme: electron from flavin derived cofactors goes to Heme domain together with, but faster than, the electron transfer from BH4; however, before the next catalytic cycle can proceed, the BH4 has to be reduced. These oxidations lead to limitation of ROS production. C: electron flowing through NOS enzyme in hypertension: there is a disruption in BH4 oxidation, leading to NOS uncoupling, reduced no production and, consequently, increased ROS.

As almost all enzymes, NOS isoforms require cofactors. Tetrahydrobiopterin (BH_4_) is one of the critical cofactors for NOS activity. In conditions that will be approached in this article such as hypertension, BH_4_ is oxidized leading to NOS uncoupling [50, 51], increased Reactive Oxygen Species (ROS) and reduced NO production due to an electron flowing through the enzyme (Fig. **2**).

## 3 NITRIC OXIDE INFLUENCES

NO acts on a number of protein targets through cell signaling. One of the most important physiological signaling is the activation of soluble guanylyl cyclase (GC) and the generation of cyclic guanosine monophosphate (cGMP) [9, 10, 52-55], especially for neurotransmission and vascular tonus functions. The transduction for the NO signaling is given by its reaction with superoxide anion (O_2_^−•^), resulting in NO inactivation and potent oxidant peroxynitrite (ONOO^−^) formation. This compound causes oxidative damage, nitration, and S-nitrosylation of biomolecules including protein, lipids, and DNA [56, 57]. These damages are primordial to the development of the components predisposing MS and its symptoms, causing respectively hypertension and pain.

### 3.1 NO Pathway in Pain

**Fig. (3):**
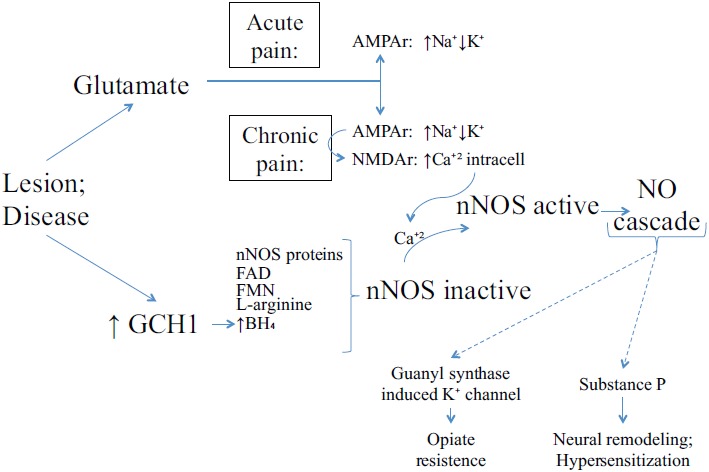
Nociceptive sensitization related to NO/ROS cascade. Neurotransmitter glutamate is secreted from the nociceptor terminal to the synaptic cleft and sensitizes AMPA receptors (AMPAr) on dorsal horn cells membrane. In long-term, the changes in membrane polarization affect the NMDA receptors (NMDAr). NMDAr sensitization allows the Ca^+2^ influx, which is essential to the neuronal Nitric Oxide Synthase (nNOS) activation. The nNOS activation is possible only if the inactive nNOS has all the cofactors (FAD, FMN, L-arginine and BH_4_) dimerized. For this, a lesion also increases the GTP cyclohydrolase (GCH1) levels, enhancing BH_4_. Ultimately, NO produced in the dorsal horn cells are released back into the synaptic cleft for closing guanyl synthase-induced K^+^ channels and for releasing Substance P. Respectively, the results are the opiate resistance in chronic pain and neural remodeling and hypersensitization.

The cardiovascular system functions reported on this article relies on blood circulation; namely, the association of angina and hypertension regarding nervous systems and blood circulation influenced by NO. In this sense, the expected NO functioning in the sensory perception of pain signaling and explanation are based on biochemical processes depicted on (Fig. **3**).

The integrity of nervous system is essential for pain protective functions. Processes responsible for converting sensory stimuli to cellular transduction, enabling the recognition and characterization of the signal, modulate the frequency, rate and extent of the sensory perception of pain. Together with the NOS isoforms, glutamate receptor is involved in some of the important signaling processes [58]. Even though these proteins are reported to be associated to the regulation of sensory perception of pain, it is still unlinked, according to resources for “protein” search at the National Center for Biotechnology Information (NCBI) database [59], if they are positive or negative regulators or even part of the modulation of pain.

The usual NO pain pathway is showed on Fig. (**3**). However, how the NOS isoforms, mainly nNOS, are related to heart pain and the mechanism by which NO can sensitize the neuronal path to trigger the perception of pain remain partially unknown. Visceral and neuropathic are the main types of pain correlated to NO and ischemia, the principal cause of heart pain. Both pathways are nociceptors-sensitized on primary afferent C fibers, where the action potential is conducted to the central nervous and to secondary afferent neurons in spinal-cord dorsal horn. Then, the signal reaches areas of the brain responsible for localization and emotional aspects of pain, respectively, through spinothalamic and spinoreticular tracts. The main difference between visceral and neuropathic pain resides on the type of stimuli for which they respond to. Smooth muscle distension or contraction, capsule stretching surrounding an organ, ischemia, necrosis or inflammatory mediators trigger visceral pain; dissimilarly, the triggers for neuropathic pain pathway are trauma, surgery, diabetes mellitus, chemotherapy, radiotherapy, infection, malignancy and ischemia in which the damage occurs directly to central or peripheral nervous system [60].

Excluding nociception stimuli from extra cardiac issues, the main etiology for angina is ischemia. Intermittent ischemia in focal myocardial regions might result into functional alterations for both efferent and afferent cardiac adrenergic, and possibly vagal, nerve fibers. Additional mechanisms such as metabolic abnormalities might also adversely affect cardiac nerve fiber function [61, 62]. Cardiac stimuli are usually unable to elicit a painful response through afferent nerve fibers due to their low-sensitiveness; however, the fibers sensitivity to cardiac stimuli is increased if there are functional alterations, such the ones caused by ischemia. Therefore, the result is a painful response and consequent greater cardiac pain perception. This process is similar to cutaneous hyperalgesia due to peripheral sympathetic fiber injury described in literature [63]. Overall, impaired myocardial circulation generates ischemia stimulating the nociceptive pathway.

In order to elucidate the relationship between NO and angina, there are numerous studies with pharmacological approaches based on biotechnology researches applying knockout NOS mice [64-82]. Several studies have related drugs based on NO mechanisms and their influences on pain or ischemic signals.

In this sense, the development of NOS inhibitors was one of the first pharmacological approaches. Regarded as a therapy, since chronic pain patients showed a significant increase in NO plasma levels in comparison with healthy individuals [83], methylene blue (MB) is the most studied drug affecting NO mechanisms [64-73, 82]. MB directly inhibits constitutive and inducible NOS [65] through cGMP accumulation avoidance by GC enzyme blockage [65, 66]. A valuable property of MB is its antioxidant effects [66]; it acts inhibiting the formation of free oxygen radicals and O_2_^−•^ by competing with molecular oxygen (O_2_). Therefore, the transfer of electrons by xanthine oxidase (XO) [68] is prevented. Studies have demonstrated MB decreasing pain levels in patients with chronic therapy-resistant neuropathic pain on the first 2 days after administration [69, 74].

Complementarily, studies using knockout mice analyzed NOS absence. In 2008, Nakata *et al*. [75] demonstrated NOS isoforms knockout mice in conditions of hypertension, hyperlipidemia, impaired glucose tolerance, insulin resistance, metabolic syndrome and presence of visceral obesity. In fact, targeted disruption of NOS genes leads to mutant mice development and allows a better understanding of NO mechanisms related to blood pressure regulation, endothelial dysfunction, response to vascular injury, response to stroke and cerebral ischemia, diet-induced atherosclerosis and cardiac contractility [76]. Results from such researches have shown the deletion of the eNOS gene led to increase blood pressure [84, 85]. Other studies analyzed the phenotype of nNOS knockout mice and noticed stomachs enlargement, several times bigger than normal size, demonstrating nNOS role in smooth muscle relaxation of the pyloric sphincter. nNOS knockout mice were also resistant to focal and global cerebral ischemia, consistent as a part of nNOS-derived NO function in cellular ischemic injury [76-80]. nNOS gene deletion has also been associated with more severe left ventricular remodeling after myocardial infarction [81].

### 3.2 NO Influences in Hypertension and Angina

According to data from the World Health Organization (WHO), cardiovascular diseases killed 17.5 million people in 2012, which are 3 in every 10 deaths globally distributed. Of these, 7.4 million people died due to ischemia and 1.1 million due to hypertensive heart diseases [86]. Both, ischemic and hypertensive heart diseases are directly influenced by coronary heart dysfunctions, which causes their usual symptom: angina [87]. In addition, one of the main prescriptions to patients with angina is glyceryl trinitrate, which belongs to the nitrates chemical group responsible for vasodilatation and consequent blood pressure decreasing.

There are many evidences on literature about the roles of NOS and cytochrome C (Cyt-C) in cardiovascular diseases [36, 75, 88-92]. The NOS influences on blood pressure and circulation vary depending on the type of the isoform. Nonetheless, Cyt-C is known to be part of NO production in strictly hypoxic conditions, such as ischemic angina. Despite these two well-studied proteins, there is a lack of evidence about what other factors are involved in such disrupted cardiovascular systems. In summary, it is possible only to correlate Cyt-C and NOS as part of the NO role in hypertension and angina.

The three NOS isoforms are active on cardiovascular system; however, the main enzymes related to hypertension and angina are eNOS and nNOS. iNOS contributes mostly to the pathophysiology of inflammatory diseases and septic shock [55, 75, 93].

NO produced by nNOS in nitrergic nerves is considered as a neurotransmitter responsible for stimulating NO-sensitive GC in its effector cells, thereby decreasing the tone of various types of smooth muscle including blood vessels [55, 94, 95]. nNOS functions include synaptic plasticity in the central nervous system (CNS), central regulation of blood pressure, smooth muscle relaxation and vasodilatation via peripheral nitrergic nerves [55]. Most importantly, nNOS plays a role in the regulation of vascular tone independent of effects from nNOS in the CNS [94, 95]. The blockage of nNOS activity in the medulla and hypothalamus causes systemic hypertension [96].

Complementarily, eNOS-derived NO dilates all types of blood vessels by stimulating soluble GC and increasing cGMP in smooth muscle cells [9, 10, 84]. NO from eNOS is a homeostatic regulator keeping blood vessels dilated, blood pressure, vasoprotection and anti-atherosclerotic effects. Although mostly expressed in endothelial cells, eNOS has also been detected in cardiomyocytes [94, 95]. Pharmacologically, vascular oxidative stress can be reduced and eNOS functionality restored with both renin- and angiotensin II- inhibitors and AT_1_ receptor blockers, and also with statins [55]. There are eNOS stimulators, the classic class of drugs for treating hypertension and myocardial infarction [35, 55, 97]. This choice for treatment is due to the powerful protective effect of eNOS-derived NO against the onset of atherogenesis. In short, NO from eNOS possesses the following effects: inhibition of platelet aggregation, vascular wall adhesion [98-100] and leucocyte adhesion to the vessel wall which are early events in the development of atherosclerosis; representing a critical factor for adaptive vascular remodeling to chronicle changes in blood flow [101]; controlling expression of genes involved in atherogenesis and angiogenesis post-ischemia [102]. The abrupt reduction on the bioavailability of eNOS-derived NO is observed after experimental myocardial infarction and in humans under heart failure condition [103, 104], contributing to impaired neovascularization [105]. Accordingly, endothelial NO can reduce the chances of angina episodes, once their effects are also correlated to ischemic-related angina.

Overall, nNOS and eNOS may have distinct roles in the physiological regulation of human microvascular tone in vivo [106]. Interestingly, low levels of nNOS have been shown in vascular smooth muscle cells as responsible to preserve some degree of vasodilatation when the predominant eNOS becomes dysfunctional [55, 107].

Hypertensive patients with metabolic syndrome and also patients with vascular diseases such as atherosclerosis show endothelial dysfunction due to reduced NO bioavailability and consequently impaired endothelium-dependent vasodilatation [108] associated with increased ROS production. There are several enzymatic systems potentially producing ROS in the vessels, including the Nicotinamide Adenine Dinucleotide Phosphate Hydrogen (NADPH) oxidases, XO mitochondrial respiratory chain, uncoupled eNOS [109] and nNOS [110-116]. Of these, NADPH oxidases are considered primary importance for ROS generation. Several isoforms of O_2_^−•^-producing NADPH oxidase are expressed in endothelial and smooth muscle cells, as well as in the adventitia layer [55].

The eNOS and nNOS produce large amounts of ROS when deprived of their critical cofactor BH_4_ or their substrate L-arginine [110-116]. An important stage of the electron transfer occurs in the Heme domain, which receives electron and enables oxygen binding. The reduction directly to the Heme domain is faster than Flavin reduction through the BH_4_ cofactor. Despite this faster process, the catalytic cycle can only proceed if BH_4_ was reduced. This difference leads to limitation of the reduced oxygen species productions by heme reduction. In hypertensive vessels Fig. (**2**), the disruption in electron flowing rather results in reduction of O_2_ at the prosthetic heme site than formation of NO [117]. The importance of the O_2_^−•^ formation is due to the BH_4_ oxidation. Some in vivo studies [118] provided a mechanism for the predisposition to atherosclerosis suggesting NADPH oxidase as the initial source of ROS leading to BH_4_ oxidation. Endothelial and vascular smooth muscle cells-derived NADPH oxidase produces superoxide, respectively in early and advanced atherosclerosis stages [119]. Despite this knowledge, Laursen *et al*. [120] described ONOO^−^ as more potent BH_4_ oxidant than O_2_^−•^ in hypertension. Indeed, myriad toxic effects of NO are recognized due to the subsequent generation of ONOO^−^ [121, 122] involved in inflammatory conditions [123, 124], neurodegenerative diseases [125, 126] and cardiovascular diseases [118, 120]. Therefore, NADPH oxidase- O_2_^−•^ may not be an oxidant as relevant in hypertension as ONOO^−^.

Similarly, the Cyt-C oxidase is a functionally competent ONOO^−^ reductase. It is suggested an enhanced NO production through a positive feedback mechanism for NO_2_^−^-derived mitochondrial NO on a Cyt-C oxidase subunit. This protein recruitment is in state-dependent hypoxia; therefore, Cyt-C functional role is in hypoxic signaling events [127].

## CONCLUSION

Manifold studies have proved or suggested the control and influences on blood pressure by NOS isoenzymes and have made correlation between NOS-NO and ischemic angina. This is because both, hypertension and ischemic angina, are part of a major MS that affect not just NO production by NOS, but also enzymes pathways, such Cyt-C, important to strictly anoxic conditions [16, 18, 128]. Due to this, it is still important to generate correlations between the many enzymes pathways already described on literature and angina for a better and more complete understanding of CVD.

In the vascular endothelium, BH_4_ mediates coupling of O_2_ reduction to heme-catalyzed L-arginine oxidation to form NO and L-citrulline [50]. In patients with MS, there is an inherently systemic inflammation and high risk of CHD [50]. The usual result is atherosclerosis in the coronary arteries leading to NADPH oxidase functioning and ROS products. Beyond ROS strengthen vascular lesions and NADPH oxidase functions, O_2_^−•^ and ONOO^−^ oxide BH_4_. Even though the pathophysiologic control of endothelial BH_4_ levels in humans is poorly known, assembling the information described in the literature databases turns possible to have a better insight about the NO roles in cardiovascular symptoms such as angina.

## Figures and Tables

**Fig. (1):**
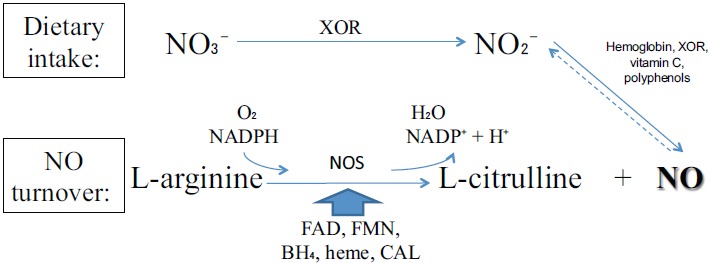
Physiological Nitric Oxide (NO) formation pathways. NO production using xanthine oxidoreductase (XOR) can be synchronized with nitric oxide synthase (NOS). NO turnover pathway is dependent on co-substracts as molecular oxygen (O_2_) and NADPH and cofactors such flavin adenine dinucleotide (FAD), flavin mononucleotide (FMN), tetrahydrobiopterin (BH_4_), heme and binding calmodulin (CAL) for NO synthesis.

## LIST OF ABBREVIATIONS

BH_4_: = Tetrahydrobiopterin
cGMP: = Cyclic guanosine monophosphate
CHD: = Coronary heart disease
CNS: = Central nervous system
Cyt-C: = Cytochrome C
eNOS: = Endothelial - Nitric Oxide Synthase
GC: = Guanylyl cyclase
iNOS: = Inducible - Nitric Oxide Synthase
MB: = Methylene Blue
mRNA: = Messenger RNA
MS: = Metabolic Syndrome
mtNOS: = Mitochondrial - Nitric Oxide Synthase
NADPH: = Nicotinamide Adenine Dinucleotide Phosphate Hydrogen
NCBI: = National Center for Biotechnology Information
nNOS: = Neuronal - Nitric Oxide Synthase
NO: = Nitric Oxide
NO_2_^−^: = Inorganic nitrite
NO_3_^−^: = Nitrate
NOS: = Nitric Oxide Synthase
O_2_: = Molecular oxygen
O_2_^−•^: = Superoxide anion
ONOO^−^: = Peroxynitrite
ROS: = Reactive Oxygen Species
XO: = Xanthine oxidase
XOR: = Xanthine oxidoreductase
